# Cyclo­hex­yl(meth­yl)ammonium {bis­[cyclo­hex­yl(meth­yl)amino]­phosphor­yl}(4-methyl­phenyl­sulfon­yl)aza­nide

**DOI:** 10.1107/S160053681103950X

**Published:** 2011-09-30

**Authors:** Mehrdad Pourayoubi, Hassan Fadaei, Atekeh Tarahhomi, Masood Parvez

**Affiliations:** a Department of Chemistry, Ferdowsi University of Mashhad, Mashhad 91779, Iran; b Department of Chemistry, University of Calgary, 2500 University Drive NW, Calgary, Alberta, Canada T2N 1N4

## Abstract

In the anion of the title salt, C_7_H_16_N^+^·C_21_H_35_N_3_O_3_PS^−^, the P and S atoms are both in distorted tetra­hedral environments and the angles at the tertiary N atoms confirm their *sp* ^2^ character. The two S=O groups are in *syn* and *gauche* conformations with respect to the phosphoryl group. In the crystal, N—H⋯O(=S) and N—H⋯O(=P) hydrogen bonds involving two anions and two cations form a centrosymmetric four-component cluster.

## Related literature

For related structures see: Yazdanbakhsh *et al.* (2009 ▶); Pourayoubi *et al.* (2011 ▶).
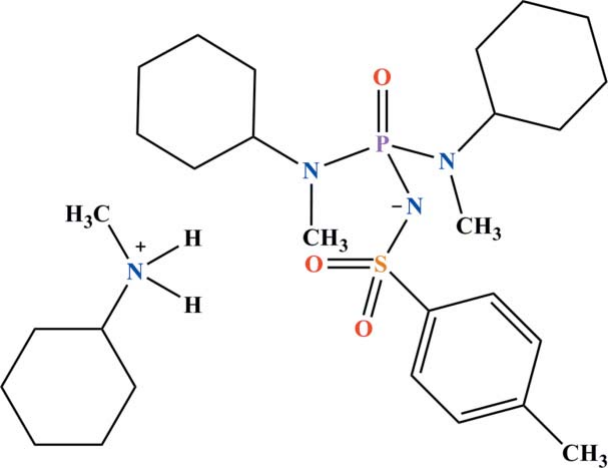


## Experimental

### 

#### Crystal data

C_7_H_16_N^+^·C_21_H_35_N_3_O_3_PS^−^
*M* *_r_* = 554.76
Triclinic, 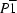

*a* = 10.6514 (5) Å
*b* = 11.5984 (5) Å
*c* = 13.5681 (6) Å
α = 103.738 (3)°
β = 97.201 (2)°
γ = 107.584 (2)°
*V* = 1516.59 (12) Å^3^
*Z* = 2
Mo *K*α radiation
μ = 0.19 mm^−1^
*T* = 173 K
0.11 × 0.10 × 0.01 mm

#### Data collection

Nonius KappaCCD diffractometer with APEXII CCD
Absorption correction: multi-scan (*SORTAV*; Blessing, 1997 ▶) *T*_min_ = 0.979, *T*_max_ = 0.998
24576 measured reflections
7401 independent reflections
4499 reflections with *I* > 2σ(*I*)
*R*_int_ = 0.160

#### Refinement

*R*[*F* ^2^ > 2σ(*F* ^2^)] = 0.085
*wR*(*F* ^2^) = 0.189
*S* = 1.07
7401 reflections
338 parameters
H-atom parameters constrained
Δρ_max_ = 0.44 e Å^−3^
Δρ_min_ = −0.48 e Å^−3^

### 

Data collection: *COLLECT* (Hooft, 1998 ▶); cell refinement: *DENZO* (Otwinowski & Minor, 1997 ▶); data reduction: *SCALEPACK* (Otwinowski & Minor, 1997 ▶); program(s) used to solve structure: *SIR92* (Altomare *et al.*, 1993 ▶); program(s) used to refine structure: *SHELXL97* (Sheldrick, 2008 ▶); molecular graphics: *Mercury* (Macrae *et al.*, 2008 ▶); software used to prepare material for publication: *enCIFer* (Allen *et al.*, 2004 ▶).

## Supplementary Material

Crystal structure: contains datablock(s) global, I. DOI: 10.1107/S160053681103950X/lh5320sup1.cif

Structure factors: contains datablock(s) I. DOI: 10.1107/S160053681103950X/lh5320Isup2.hkl

Supplementary material file. DOI: 10.1107/S160053681103950X/lh5320Isup3.cml

Additional supplementary materials:  crystallographic information; 3D view; checkCIF report

## Figures and Tables

**Table 1:** Hydrogen-bond geometry (Å, °)

*D*—H⋯*A*	*D*—H	H⋯*A*	*D*⋯*A*	*D*—H⋯*A*
N4—H42⋯O2	0.92	1.98	2.864 (4)	160
N4—H41⋯O1^i^	0.92	1.76	2.648 (4)	163

Symmetry code: (i) 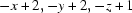.

**Fig. 1.**
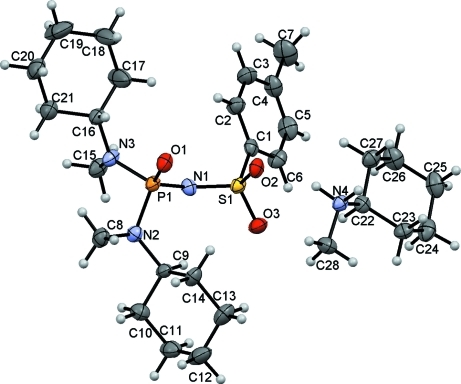
The molecular structure of the title salt. Displacement ellipsoids are given at 50% probability level and H atoms are drawn as small spheres of arbitrary radii.

**Fig. 2.**
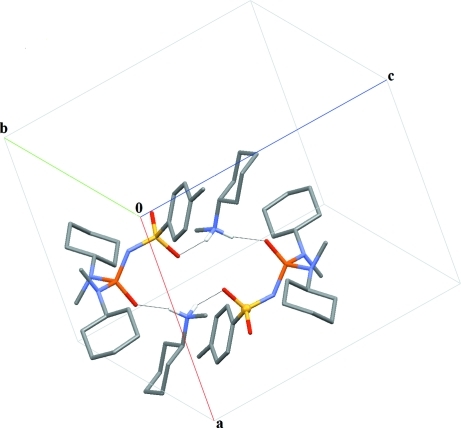
A view of the hydrogen-bonded centrosymmetric cluster, containing two phosphonic triamide anions and two N-methylcyclohexyl ammonium cations. The N—H···O hydrogen bonds are shown as dotted lines. The H atoms not involved in hydrogen bonding have been omitted for the sake of clarity.

## supplementary crystallographic information

### Comment

The proton transfer compound, {C_6_H_11_NH_2_CH_3_}^+^.{4-CH_3_C_6_H_4_S(O)_2_NP(O)[N(CH_3_)(C_6_H_11_)]_2_}^-^, contains an *N*-methylcyclohexyl ammonium cation and a deprotonated *N*,*N'*-dicyclohexyl-*N*,*N'* -dimethyl-*N"*-(*p*-toluenesulfonyl)phosphoric triamide (Fig. 1).

The P═O and P—N bond lengths and the P—N—C bond angles are comparable to those in a similar previously reported proton transfer compound, {C_6_H_11_NH_2_CH_3_}^+^{CF_3_C(O)NP(O)[N(CH_3_)(C_6_H_11_)]_2_}^-^ (Yazdanbakhsh *et al.*, 2009).

The P—N1 bond (1.608 (3) Å) is shorter than the P—N2 (1.668 (3) Å) and P—N3 (1.654 (3) Å) bonds and the P1—N1—S1 bond angle is 133.1 (2)°. The S═O bond lengths of 1.449 (3) Å & 1.466 (3) Å are standard for sulfonamide compounds (Pourayoubi *et al.*, 2011).

Each of the phosphorus and sulfur atoms has a distorted tetrahedral configuration. The bond angles around the P and S atoms are in the range of 103.00 (16)° to 118.19 (15)° and 103.78 (17)° to 114.45 (16)°, respectively.

In the crystal, two phosphonic triamide anions and two *N*-methylcyclohexyl ammonium cations are hydrogen-bonded into a centrosymmetric four-component cluster *via* N—H···O(═S) and N—H···O(═P) hydrogen bonds (Fig. 2).

### Experimental

Synthesis of 4-CH_3_C_6_H_4_S(O)_2_NHP(O)Cl_2_: 4-CH_3_C_6_H_4_S(O)_2_NHP(O)Cl_2_ was synthesized from the reaction between phosphorus pentachloride (19 mmol) and *p*-toluenesulfonamide (19 mmol) in dry CCl_4_ (20 ml) at 353 K (4 h) and then treated with formic acid (19 mmol) at ice bath temperature.

Synthesis of the title salt: To a solution of 4-CH_3_C_6_H_4_S(O)_2_NHP(O)Cl_2_ (1.9 mmol) in dry chloroform (20 ml), a solution of *N*-methylcyclohexylamine (9.5 mmol) in dry chloroform (10 ml) was added at 273 K. After 4 h stirring, the solvent was removed and the obtained product was washed with deionized water and recrystallized from methanol at room temperature.

### Refinement

H-atoms were included in geometrically idealized positions with C—H = 0.95 - 1.00 Å and N—H = 0.92 Å and included in the refinement with *U*_iso_(H) = 1.2*U*_eq_(C/N).

### Crystal data

**Table:**

C_7_H_16_N^+^·C_21_H_35_N_3_O_3_PS^−^	*Z* = 2
*M**_r_* = 554.76	*F*(000) = 604
Triclinic, *P*1	*D*_x_ = 1.215 Mg m^−^^3^
Hall symbol: -P 1	Mo *K*α radiation, λ = 0.71073 Å
*a* = 10.6514 (5) Å	Cell parameters from 4260 reflections
*b* = 11.5984 (5) Å	θ = 1.6–28.3°
*c* = 13.5681 (6) Å	µ = 0.19 mm^−^^1^
α = 103.738 (3)°	*T* = 173 K
β = 97.201 (2)°	Prism, colorless
γ = 107.584 (2)°	0.11 × 0.10 × 0.01 mm
*V* = 1516.59 (12) Å^3^	

### Data collection

**Table:**

Nonius KappaCCD diffractometer with APEXII CCD	7401 independent reflections
Radiation source: fine-focus sealed tube	4499 reflections with *I* > 2σ(*I*)
graphite	*R*_int_ = 0.160
ω and φ scans	θ_max_ = 28.3°, θ_min_ = 1.6°
Absorption correction: multi-scan (*SORTAV*; Blessing, 1997)	*h* = −14→14
*T*_min_ = 0.979, *T*_max_ = 0.998	*k* = −15→15
24576 measured reflections	*l* = −17→18

### Refinement

**Table:**

Refinement on *F*^2^	Primary atom site location: structure-invariant direct methods
Least-squares matrix: full	Secondary atom site location: difference Fourier map
*R*[*F*^2^ > 2σ(*F*^2^)] = 0.085	Hydrogen site location: inferred from neighbouring sites
*wR*(*F*^2^) = 0.189	H-atom parameters constrained
*S* = 1.07	*w* = 1/[σ^2^(*F*_o_^2^) + (0.*P*)^2^ + 3.380*P*] where *P* = (*F*_o_^2^ + 2*F*_c_^2^)/3
7401 reflections	(Δ/σ)_max_ = 0.003
338 parameters	Δρ_max_ = 0.44 e Å^−^^3^
0 restraints	Δρ_min_ = −0.48 e Å^−^^3^

### Special details

**Table:**

Geometry. All e.s.d.'s (except the e.s.d. in the dihedral angle between two l.s. planes) are estimated using the full covariance matrix. The cell e.s.d.'s are taken into account individually in the estimation of e.s.d.'s in distances, angles and torsion angles; correlations between e.s.d.'s in cell parameters are only used when they are defined by crystal symmetry. An approximate (isotropic) treatment of cell e.s.d.'s is used for estimating e.s.d.'s involving l.s. planes.
Refinement. Refinement of *F*^2^ against ALL reflections. The weighted *R*-factor *wR* and goodness of fit *S* are based on *F*^2^, conventional *R*-factors *R* are based on *F*, with *F* set to zero for negative *F*^2^. The threshold expression of *F*^2^ > σ(*F*^2^) is used only for calculating *R*-factors(gt) *etc*. and is not relevant to the choice of reflections for refinement. *R*-factors based on *F*^2^ are statistically about twice as large as those based on *F*, and *R*- factors based on ALL data will be even larger.

### Fractional atomic coordinates and isotropic or equivalent isotropic displacement parameters (Å^2^)

**Table:**

	*x*	*y*	*z*	*U*_iso_*/*U*_eq_	
S1	0.70329 (9)	0.92657 (8)	0.36570 (7)	0.0249 (2)	
P1	0.79120 (10)	0.92027 (9)	0.17273 (7)	0.0240 (2)	
O1	0.9263 (3)	0.9142 (2)	0.20889 (19)	0.0309 (6)	
O2	0.8350 (2)	0.9315 (2)	0.41750 (19)	0.0292 (6)	
O3	0.6634 (3)	1.0300 (2)	0.4178 (2)	0.0337 (6)	
N1	0.6862 (3)	0.9065 (3)	0.2483 (2)	0.0254 (7)	
N2	0.7991 (3)	1.0530 (3)	0.1422 (2)	0.0271 (7)	
N3	0.7199 (3)	0.8094 (3)	0.0606 (2)	0.0291 (7)	
C1	0.5849 (4)	0.7865 (3)	0.3761 (3)	0.0261 (8)	
C2	0.5958 (4)	0.6703 (4)	0.3313 (3)	0.0355 (10)	
H2	0.6642	0.6656	0.2934	0.043*	
C3	0.5076 (5)	0.5618 (4)	0.3417 (3)	0.0412 (10)	
H3	0.5165	0.4828	0.3111	0.049*	
C4	0.4056 (4)	0.5647 (4)	0.3959 (3)	0.0374 (10)	
C5	0.3963 (4)	0.6819 (4)	0.4392 (3)	0.0415 (11)	
H5	0.3271	0.6866	0.4762	0.050*	
C6	0.4843 (4)	0.7921 (4)	0.4303 (3)	0.0334 (9)	
H6	0.4759	0.8713	0.4612	0.040*	
C7	0.3111 (5)	0.4448 (5)	0.4076 (4)	0.0595 (14)	
H7A	0.2376	0.4627	0.4376	0.071*	
H7B	0.3604	0.4119	0.4534	0.071*	
H7C	0.2738	0.3819	0.3393	0.071*	
C8	0.8916 (4)	1.0830 (4)	0.0727 (3)	0.0364 (10)	
H8A	0.8660	1.1381	0.0356	0.044*	
H8B	0.8867	1.0046	0.0225	0.044*	
H8C	0.9840	1.1263	0.1137	0.044*	
C9	0.7912 (4)	1.1622 (3)	0.2199 (3)	0.0263 (8)	
H9	0.7191	1.1282	0.2566	0.032*	
C10	0.7481 (4)	1.2506 (4)	0.1686 (3)	0.0350 (9)	
H10A	0.6627	1.2024	0.1168	0.042*	
H10B	0.8175	1.2868	0.1316	0.042*	
C11	0.7284 (5)	1.3574 (4)	0.2483 (4)	0.0432 (11)	
H11A	0.6547	1.3218	0.2820	0.052*	
H11B	0.7025	1.4149	0.2130	0.052*	
C12	0.8583 (5)	1.4315 (4)	0.3306 (4)	0.0530 (13)	
H12A	0.9299	1.4733	0.2977	0.064*	
H12B	0.8430	1.4979	0.3836	0.064*	
C13	0.9034 (5)	1.3434 (4)	0.3824 (4)	0.0473 (12)	
H13A	0.8356	1.3084	0.4210	0.057*	
H13B	0.9899	1.3921	0.4329	0.057*	
C14	0.9211 (4)	1.2349 (4)	0.3028 (3)	0.0340 (9)	
H14A	0.9450	1.1768	0.3384	0.041*	
H14B	0.9958	1.2691	0.2694	0.041*	
C15	0.5777 (4)	0.7821 (4)	0.0194 (3)	0.0363 (9)	
H15A	0.5591	0.7524	−0.0566	0.044*	
H15B	0.5555	0.8590	0.0415	0.044*	
H15C	0.5228	0.7164	0.0457	0.044*	
C16	0.7784 (4)	0.7114 (3)	0.0248 (3)	0.0295 (8)	
H16	0.8779	0.7504	0.0531	0.035*	
C17	0.7285 (7)	0.5975 (5)	0.0641 (5)	0.075 (2)	
H17A	0.6301	0.5556	0.0371	0.089*	
H17B	0.7453	0.6253	0.1409	0.089*	
C18	0.7997 (9)	0.5038 (6)	0.0296 (5)	0.096 (3)	
H18A	0.8963	0.5427	0.0640	0.115*	
H18B	0.7609	0.4279	0.0521	0.115*	
C19	0.7873 (6)	0.4648 (4)	−0.0850 (5)	0.0689 (17)	
H19A	0.6922	0.4137	−0.1192	0.083*	
H19B	0.8429	0.4113	−0.1027	0.083*	
C20	0.8317 (5)	0.5767 (4)	−0.1252 (4)	0.0472 (12)	
H20A	0.8147	0.5476	−0.2019	0.057*	
H20B	0.9299	0.6211	−0.0987	0.057*	
C21	0.7576 (5)	0.6686 (4)	−0.0922 (3)	0.0388 (10)	
H21A	0.7916	0.7429	−0.1178	0.047*	
H21B	0.6601	0.6267	−0.1233	0.047*	
N4	0.9037 (3)	1.0830 (3)	0.6297 (2)	0.0253 (7)	
H41	0.9713	1.0780	0.6760	0.030*	
H42	0.8916	1.0232	0.5677	0.030*	
C22	0.7756 (4)	1.0497 (3)	0.6696 (3)	0.0274 (8)	
H22	0.7016	1.0541	0.6190	0.033*	
C23	0.7914 (4)	1.1430 (4)	0.7748 (3)	0.0331 (9)	
H23A	0.8685	1.1440	0.8245	0.040*	
H23B	0.8105	1.2291	0.7674	0.040*	
C24	0.6636 (4)	1.1058 (4)	0.8168 (3)	0.0411 (10)	
H24A	0.6785	1.1633	0.8873	0.049*	
H24B	0.5894	1.1158	0.7717	0.049*	
C25	0.6227 (5)	0.9703 (4)	0.8216 (3)	0.0453 (11)	
H25A	0.5353	0.9476	0.8433	0.054*	
H25B	0.6909	0.9628	0.8740	0.054*	
C26	0.6099 (4)	0.8787 (4)	0.7162 (3)	0.0426 (11)	
H26A	0.5894	0.7921	0.7226	0.051*	
H26B	0.5344	0.8788	0.6656	0.051*	
C27	0.7398 (4)	0.9157 (4)	0.6762 (3)	0.0346 (9)	
H27A	0.8140	0.9089	0.7236	0.042*	
H27B	0.7278	0.8572	0.6067	0.042*	
C28	0.9490 (4)	1.2093 (4)	0.6132 (3)	0.0357 (9)	
H28A	0.8743	1.2203	0.5710	0.043*	
H28B	1.0240	1.2167	0.5771	0.043*	
H28C	0.9790	1.2745	0.6805	0.043*	

### Atomic displacement parameters (Å^2^)

**Table:**

	*U*^11^	*U*^22^	*U*^33^	*U*^12^	*U*^13^	*U*^23^
S1	0.0237 (5)	0.0258 (5)	0.0231 (5)	0.0096 (4)	0.0021 (4)	0.0033 (4)
P1	0.0224 (5)	0.0250 (5)	0.0216 (5)	0.0097 (4)	−0.0008 (4)	0.0024 (4)
O1	0.0244 (14)	0.0409 (15)	0.0249 (14)	0.0162 (12)	−0.0014 (11)	0.0021 (12)
O2	0.0231 (14)	0.0383 (15)	0.0215 (13)	0.0109 (12)	−0.0012 (11)	0.0030 (11)
O3	0.0385 (17)	0.0281 (14)	0.0351 (15)	0.0163 (12)	0.0072 (13)	0.0040 (12)
N1	0.0211 (16)	0.0304 (16)	0.0250 (16)	0.0092 (13)	0.0027 (13)	0.0094 (13)
N2	0.0262 (17)	0.0276 (16)	0.0267 (16)	0.0089 (13)	0.0067 (14)	0.0065 (13)
N3	0.0247 (17)	0.0291 (17)	0.0270 (17)	0.0129 (14)	−0.0058 (14)	−0.0023 (14)
C1	0.0237 (19)	0.034 (2)	0.0203 (18)	0.0117 (16)	−0.0006 (15)	0.0075 (16)
C2	0.041 (3)	0.029 (2)	0.037 (2)	0.0111 (18)	0.018 (2)	0.0093 (18)
C3	0.052 (3)	0.030 (2)	0.040 (2)	0.013 (2)	0.012 (2)	0.0072 (19)
C4	0.030 (2)	0.042 (2)	0.035 (2)	0.0017 (18)	0.0061 (19)	0.015 (2)
C5	0.027 (2)	0.059 (3)	0.044 (3)	0.014 (2)	0.017 (2)	0.021 (2)
C6	0.026 (2)	0.039 (2)	0.036 (2)	0.0147 (18)	0.0085 (18)	0.0075 (18)
C7	0.049 (3)	0.056 (3)	0.061 (3)	−0.005 (2)	0.011 (3)	0.024 (3)
C8	0.042 (3)	0.036 (2)	0.031 (2)	0.0106 (19)	0.0165 (19)	0.0087 (18)
C9	0.031 (2)	0.0207 (18)	0.0281 (19)	0.0106 (15)	0.0094 (17)	0.0048 (15)
C10	0.035 (2)	0.034 (2)	0.038 (2)	0.0140 (18)	0.0060 (19)	0.0136 (19)
C11	0.055 (3)	0.038 (2)	0.052 (3)	0.027 (2)	0.025 (2)	0.019 (2)
C12	0.068 (4)	0.029 (2)	0.060 (3)	0.016 (2)	0.028 (3)	0.003 (2)
C13	0.052 (3)	0.039 (3)	0.038 (2)	0.013 (2)	0.006 (2)	−0.005 (2)
C14	0.036 (2)	0.029 (2)	0.028 (2)	0.0079 (17)	0.0038 (18)	−0.0011 (17)
C15	0.030 (2)	0.036 (2)	0.035 (2)	0.0099 (18)	0.0003 (18)	0.0016 (18)
C16	0.030 (2)	0.030 (2)	0.027 (2)	0.0127 (17)	0.0027 (17)	0.0039 (16)
C17	0.139 (6)	0.066 (4)	0.074 (4)	0.069 (4)	0.077 (4)	0.049 (3)
C18	0.194 (8)	0.079 (4)	0.089 (5)	0.103 (5)	0.088 (5)	0.060 (4)
C19	0.089 (4)	0.035 (3)	0.089 (4)	0.027 (3)	0.050 (4)	0.006 (3)
C20	0.060 (3)	0.044 (3)	0.036 (2)	0.019 (2)	0.020 (2)	0.003 (2)
C21	0.044 (3)	0.039 (2)	0.032 (2)	0.015 (2)	0.010 (2)	0.0062 (19)
N4	0.0235 (16)	0.0256 (16)	0.0245 (16)	0.0090 (13)	0.0020 (13)	0.0036 (13)
C22	0.0214 (19)	0.031 (2)	0.0277 (19)	0.0089 (16)	0.0008 (16)	0.0072 (16)
C23	0.032 (2)	0.031 (2)	0.038 (2)	0.0147 (17)	0.0093 (18)	0.0076 (18)
C24	0.036 (2)	0.056 (3)	0.034 (2)	0.023 (2)	0.010 (2)	0.008 (2)
C25	0.033 (2)	0.065 (3)	0.039 (3)	0.011 (2)	0.013 (2)	0.022 (2)
C26	0.031 (2)	0.047 (3)	0.044 (3)	0.0015 (19)	0.005 (2)	0.018 (2)
C27	0.033 (2)	0.032 (2)	0.033 (2)	0.0078 (17)	0.0027 (18)	0.0044 (18)
C28	0.039 (2)	0.031 (2)	0.041 (2)	0.0134 (18)	0.007 (2)	0.0161 (19)

### Geometric parameters (Å, °)

**Table:**

S1—O3	1.449 (3)	C14—H14B	0.9900
S1—O2	1.466 (3)	C15—H15A	0.9800
S1—N1	1.533 (3)	C15—H15B	0.9800
S1—C1	1.779 (4)	C15—H15C	0.9800
P1—O1	1.489 (3)	C16—C21	1.512 (5)
P1—N1	1.608 (3)	C16—C17	1.515 (6)
P1—N3	1.654 (3)	C16—H16	1.0000
P1—N2	1.668 (3)	C17—C18	1.523 (7)
N2—C8	1.471 (5)	C17—H17A	0.9900
N2—C9	1.475 (4)	C17—H17B	0.9900
N3—C15	1.456 (5)	C18—C19	1.489 (8)
N3—C16	1.469 (5)	C18—H18A	0.9900
C1—C6	1.382 (5)	C18—H18B	0.9900
C1—C2	1.386 (5)	C19—C20	1.495 (6)
C2—C3	1.375 (5)	C19—H19A	0.9900
C2—H2	0.9500	C19—H19B	0.9900
C3—C4	1.391 (6)	C20—C21	1.526 (6)
C3—H3	0.9500	C20—H20A	0.9900
C4—C5	1.385 (6)	C20—H20B	0.9900
C4—C7	1.506 (6)	C21—H21A	0.9900
C5—C6	1.381 (6)	C21—H21B	0.9900
C5—H5	0.9500	N4—C28	1.478 (4)
C6—H6	0.9500	N4—C22	1.506 (5)
C7—H7A	0.9800	N4—H41	0.9200
C7—H7B	0.9800	N4—H42	0.9200
C7—H7C	0.9800	C22—C27	1.511 (5)
C8—H8A	0.9800	C22—C23	1.528 (5)
C8—H8B	0.9800	C22—H22	1.0000
C8—H8C	0.9800	C23—C24	1.523 (6)
C9—C10	1.512 (5)	C23—H23A	0.9900
C9—C14	1.532 (5)	C23—H23B	0.9900
C9—H9	1.0000	C24—C25	1.519 (6)
C10—C11	1.525 (5)	C24—H24A	0.9900
C10—H10A	0.9900	C24—H24B	0.9900
C10—H10B	0.9900	C25—C26	1.528 (6)
C11—C12	1.529 (7)	C25—H25A	0.9900
C11—H11A	0.9900	C25—H25B	0.9900
C11—H11B	0.9900	C26—C27	1.527 (6)
C12—C13	1.524 (7)	C26—H26A	0.9900
C12—H12A	0.9900	C26—H26B	0.9900
C12—H12B	0.9900	C27—H27A	0.9900
C13—C14	1.527 (5)	C27—H27B	0.9900
C13—H13A	0.9900	C28—H28A	0.9800
C13—H13B	0.9900	C28—H28B	0.9800
C14—H14A	0.9900	C28—H28C	0.9800
			
O3—S1—O2	112.95 (16)	N3—C15—H15C	109.5
O3—S1—N1	112.97 (16)	H15A—C15—H15C	109.5
O2—S1—N1	114.45 (16)	H15B—C15—H15C	109.5
O3—S1—C1	106.07 (17)	N3—C16—C21	112.2 (3)
O2—S1—C1	105.50 (16)	N3—C16—C17	113.7 (3)
N1—S1—C1	103.78 (17)	C21—C16—C17	109.6 (4)
O1—P1—N1	118.19 (15)	N3—C16—H16	107.0
O1—P1—N3	107.98 (15)	C21—C16—H16	107.0
N1—P1—N3	108.32 (16)	C17—C16—H16	107.0
O1—P1—N2	113.12 (16)	C16—C17—C18	110.8 (4)
N1—P1—N2	105.11 (15)	C16—C17—H17A	109.5
N3—P1—N2	103.00 (16)	C18—C17—H17A	109.5
S1—N1—P1	133.1 (2)	C16—C17—H17B	109.5
C8—N2—C9	115.9 (3)	C18—C17—H17B	109.5
C8—N2—P1	113.6 (2)	H17A—C17—H17B	108.1
C9—N2—P1	120.2 (2)	C19—C18—C17	112.5 (5)
C15—N3—C16	118.1 (3)	C19—C18—H18A	109.1
C15—N3—P1	117.7 (3)	C17—C18—H18A	109.1
C16—N3—P1	120.2 (2)	C19—C18—H18B	109.1
C6—C1—C2	119.6 (4)	C17—C18—H18B	109.1
C6—C1—S1	120.9 (3)	H18A—C18—H18B	107.8
C2—C1—S1	119.5 (3)	C18—C19—C20	111.6 (4)
C3—C2—C1	120.0 (4)	C18—C19—H19A	109.3
C3—C2—H2	120.0	C20—C19—H19A	109.3
C1—C2—H2	120.0	C18—C19—H19B	109.3
C2—C3—C4	121.7 (4)	C20—C19—H19B	109.3
C2—C3—H3	119.2	H19A—C19—H19B	108.0
C4—C3—H3	119.2	C19—C20—C21	111.6 (4)
C5—C4—C3	117.2 (4)	C19—C20—H20A	109.3
C5—C4—C7	122.0 (4)	C21—C20—H20A	109.3
C3—C4—C7	120.8 (4)	C19—C20—H20B	109.3
C6—C5—C4	122.1 (4)	C21—C20—H20B	109.3
C6—C5—H5	118.9	H20A—C20—H20B	108.0
C4—C5—H5	118.9	C16—C21—C20	110.4 (4)
C5—C6—C1	119.5 (4)	C16—C21—H21A	109.6
C5—C6—H6	120.3	C20—C21—H21A	109.6
C1—C6—H6	120.3	C16—C21—H21B	109.6
C4—C7—H7A	109.5	C20—C21—H21B	109.6
C4—C7—H7B	109.5	H21A—C21—H21B	108.1
H7A—C7—H7B	109.5	C28—N4—C22	115.7 (3)
C4—C7—H7C	109.5	C28—N4—H41	108.3
H7A—C7—H7C	109.5	C22—N4—H41	108.3
H7B—C7—H7C	109.5	C28—N4—H42	108.3
N2—C8—H8A	109.5	C22—N4—H42	108.3
N2—C8—H8B	109.5	H41—N4—H42	107.4
H8A—C8—H8B	109.5	N4—C22—C27	108.8 (3)
N2—C8—H8C	109.5	N4—C22—C23	110.7 (3)
H8A—C8—H8C	109.5	C27—C22—C23	111.4 (3)
H8B—C8—H8C	109.5	N4—C22—H22	108.6
N2—C9—C10	111.3 (3)	C27—C22—H22	108.6
N2—C9—C14	113.7 (3)	C23—C22—H22	108.6
C10—C9—C14	110.6 (3)	C24—C23—C22	110.5 (3)
N2—C9—H9	106.9	C24—C23—H23A	109.5
C10—C9—H9	106.9	C22—C23—H23A	109.5
C14—C9—H9	106.9	C24—C23—H23B	109.5
C9—C10—C11	111.2 (3)	C22—C23—H23B	109.5
C9—C10—H10A	109.4	H23A—C23—H23B	108.1
C11—C10—H10A	109.4	C25—C24—C23	112.0 (3)
C9—C10—H10B	109.4	C25—C24—H24A	109.2
C11—C10—H10B	109.4	C23—C24—H24A	109.2
H10A—C10—H10B	108.0	C25—C24—H24B	109.2
C10—C11—C12	110.4 (4)	C23—C24—H24B	109.2
C10—C11—H11A	109.6	H24A—C24—H24B	107.9
C12—C11—H11A	109.6	C24—C25—C26	111.1 (3)
C10—C11—H11B	109.6	C24—C25—H25A	109.4
C12—C11—H11B	109.6	C26—C25—H25A	109.4
H11A—C11—H11B	108.1	C24—C25—H25B	109.4
C13—C12—C11	110.4 (4)	C26—C25—H25B	109.4
C13—C12—H12A	109.6	H25A—C25—H25B	108.0
C11—C12—H12A	109.6	C27—C26—C25	111.1 (3)
C13—C12—H12B	109.6	C27—C26—H26A	109.4
C11—C12—H12B	109.6	C25—C26—H26A	109.4
H12A—C12—H12B	108.1	C27—C26—H26B	109.4
C12—C13—C14	111.5 (4)	C25—C26—H26B	109.4
C12—C13—H13A	109.3	H26A—C26—H26B	108.0
C14—C13—H13A	109.3	C22—C27—C26	110.2 (3)
C12—C13—H13B	109.3	C22—C27—H27A	109.6
C14—C13—H13B	109.3	C26—C27—H27A	109.6
H13A—C13—H13B	108.0	C22—C27—H27B	109.6
C13—C14—C9	110.6 (3)	C26—C27—H27B	109.6
C13—C14—H14A	109.5	H27A—C27—H27B	108.1
C9—C14—H14A	109.5	N4—C28—H28A	109.5
C13—C14—H14B	109.5	N4—C28—H28B	109.5
C9—C14—H14B	109.5	H28A—C28—H28B	109.5
H14A—C14—H14B	108.1	N4—C28—H28C	109.5
N3—C15—H15A	109.5	H28A—C28—H28C	109.5
N3—C15—H15B	109.5	H28B—C28—H28C	109.5
H15A—C15—H15B	109.5		
			
O3—S1—N1—P1	114.9 (3)	C8—N2—C9—C10	58.5 (4)
O2—S1—N1—P1	−16.2 (3)	P1—N2—C9—C10	−158.7 (3)
C1—S1—N1—P1	−130.6 (3)	C8—N2—C9—C14	−67.3 (4)
O1—P1—N1—S1	21.6 (3)	P1—N2—C9—C14	75.6 (4)
N3—P1—N1—S1	144.7 (3)	N2—C9—C10—C11	175.2 (3)
N2—P1—N1—S1	−105.7 (3)	C14—C9—C10—C11	−57.4 (4)
O1—P1—N2—C8	49.5 (3)	C9—C10—C11—C12	57.8 (5)
N1—P1—N2—C8	179.8 (3)	C10—C11—C12—C13	−56.6 (5)
N3—P1—N2—C8	−66.8 (3)	C11—C12—C13—C14	56.2 (5)
O1—P1—N2—C9	−94.2 (3)	C12—C13—C14—C9	−55.7 (5)
N1—P1—N2—C9	36.2 (3)	N2—C9—C14—C13	−178.0 (3)
N3—P1—N2—C9	149.5 (3)	C10—C9—C14—C13	55.9 (4)
O1—P1—N3—C15	169.1 (3)	C15—N3—C16—C21	55.4 (5)
N1—P1—N3—C15	40.0 (3)	P1—N3—C16—C21	−147.6 (3)
N2—P1—N3—C15	−71.0 (3)	C15—N3—C16—C17	−69.7 (5)
O1—P1—N3—C16	12.0 (3)	P1—N3—C16—C17	87.4 (4)
N1—P1—N3—C16	−117.1 (3)	N3—C16—C17—C18	−176.5 (5)
N2—P1—N3—C16	131.9 (3)	C21—C16—C17—C18	57.0 (6)
O3—S1—C1—C6	−2.8 (4)	C16—C17—C18—C19	−55.0 (8)
O2—S1—C1—C6	117.3 (3)	C17—C18—C19—C20	53.1 (8)
N1—S1—C1—C6	−122.0 (3)	C18—C19—C20—C21	−54.1 (7)
O3—S1—C1—C2	178.9 (3)	N3—C16—C21—C20	174.4 (3)
O2—S1—C1—C2	−61.1 (3)	C17—C16—C21—C20	−58.3 (5)
N1—S1—C1—C2	59.6 (3)	C19—C20—C21—C16	57.2 (5)
C6—C1—C2—C3	−0.5 (6)	C28—N4—C22—C27	176.8 (3)
S1—C1—C2—C3	177.9 (3)	C28—N4—C22—C23	−60.4 (4)
C1—C2—C3—C4	0.4 (7)	N4—C22—C23—C24	−177.7 (3)
C2—C3—C4—C5	0.1 (7)	C27—C22—C23—C24	−56.5 (4)
C2—C3—C4—C7	−179.2 (4)	C22—C23—C24—C25	54.5 (5)
C3—C4—C5—C6	−0.5 (7)	C23—C24—C25—C26	−54.2 (5)
C7—C4—C5—C6	178.8 (4)	C24—C25—C26—C27	55.2 (5)
C4—C5—C6—C1	0.4 (6)	N4—C22—C27—C26	−179.8 (3)
C2—C1—C6—C5	0.1 (6)	C23—C22—C27—C26	57.8 (4)
S1—C1—C6—C5	−178.3 (3)	C25—C26—C27—C22	−57.0 (5)

### Hydrogen-bond geometry (Å, °)

**Table:**

*D*—H···*A*	*D*—H	H···*A*	*D*···*A*	*D*—H···*A*
N4—H42···O2	0.92	1.98	2.864 (4)	160.
N4—H41···O1^i^	0.92	1.76	2.648 (4)	163.

Symmetry codes: (i) −*x*+2, −*y*+2, −*z*+1.
